# Survival After Breast Conservation vs Mastectomy Adjusted for Comorbidity and Socioeconomic Status: A Swedish National 6-Year Follow-up of 48 986 Women

**DOI:** 10.1001/jamasurg.2021.1438

**Published:** 2021-05-05

**Authors:** Jana de Boniface, Robert Szulkin, Anna L. V. Johansson

**Affiliations:** 1 Department of Surgery, Capio St Göran’s Hospital, Stockholm, Sweden; 2 Department of Molecular Medicine and Surgery, Karolinska Institutet, Stockholm, Sweden; 3 SDS Life Science, Danderyd, Sweden; 4 Department of Medical Epidemiology and Biostatistics, Karolinska Institutet, Stockholm, Sweden; 5 Cancer Registry of Norway, Oslo, Norway

## Abstract

**Importance:**

Cohort studies show better survival after breast-conserving surgery (BCS) with postoperative radiotherapy (RT) than after mastectomy (Mx) without RT. It remains unclear whether this is an independent effect or a consequence of selection bias.

**Objective:**

To determine whether the reported survival benefit of breast conservation is eliminated by adjustment for 2 pivotal confounders, comorbidity and socioeconomic status.

**Design, Setting, and Participants:**

Cohort study using prospectively collected national data. Swedish public health care; nationwide clinical data from the National Breast Cancer Quality Register, comorbidity data from Patient Registers at the National Board of Health and Welfare, and individual-level education and income data from Statistics Sweden. The cohort included all women diagnosed as having primary invasive T1-2 N0-2 breast cancer and undergoing breast surgery in Sweden from 2008 to 2017. Data were analyzed between August 19, 2020, and November 12, 2020.

**Exposures:**

Locoregional treatment comparing 3 groups: breast-conserving surgery with radiotherapy (BCS+RT), mastectomy without radiotherapy (Mx-RT), and mastectomy with radiotherapy (Mx+RT).

**Main Outcomes and Measures:**

Overall survival (OS) and breast cancer–specific survival (BCSS). Main outcomes were determined before initiation of data retrieval.

**Results:**

Among 48 986 women, 29 367 (59.9%) had BCS+RT, 12413 (25.3%) had Mx-RT, and 7206 (14.7%) had Mx+RT. Median follow-up was 6.28 years (range, 0.01-11.70). All-cause death occurred in 6573 cases, with death caused by breast cancer in 2313 cases; 5-year OS was 91.1% (95% CI, 90.8-91.3) and BCSS was 96.3% (95% CI, 96.1-96.4). Apart from expected differences in clinical parameters, women receiving Mx-RT were older, had a lower level of education, and lower income. Both Mx groups had a higher comorbidity burden irrespective of RT. After stepwise adjustment for all covariates, OS and BCSS were significantly worse after Mx-RT (hazard ratio [HR], 1.79; 95% CI, 1.66-1.92 and HR, 1.66; 95% CI, 1.45-1.90, respectively) and Mx+RT (HR, 1.24; 95% CI, 1.13-1.37 and HR, 1.26; 95% CI, 1.08-1.46, respectively) than after BCS+RT.

**Conclusions and Relevance:**

Despite adjustment for previously unmeasured confounders, BCS+RT yielded better survival than Mx irrespective of RT. If both interventions are valid options, mastectomy should not be regarded as equal to breast conservation.

## Key Points

**Question:**

Does breast conservation offer a survival benefit compared with mastectomy when results are adjusted for main confounders such as comorbidity and socioeconomic status?

**Findings:**

In this large cohort study based on prospectively collected national data from 48 986 patients with breast cancer, overall and breast cancer–specific survival were significantly better after breast-conserving surgery followed by radiotherapy than after mastectomy with or without radiotherapy despite stepwise adjustment for tumor characteristics, treatment, demographics, comorbidity, and socioeconomic background.

**Meaning:**

Breast conservation seems to offer a survival benefit independent of measured confounders and should be given priority if both breast conservation and mastectomy are valid options.

## Teaser

This study determines whether the reported survival benefit of breast conservation is eliminated by adjustment for 2 pivotal confounders, comorbidity and socioeconomic status.

## Introduction

Since the publication of key trials^1,2^ confirming the oncological equivalence of breast-conserving surgery (BCS) followed by adjuvant radiotherapy (RT) and mastectomy (Mx), BCS is recommended for patients with early breast cancer. Additionally, in case of advanced lymph node involvement, Mx does not confer any survival benefit.^3^ The same is true for the younger breast cancer population and in specific subtypes such as triple-negative breast cancer (TNBC).^4,5,6^ Recently, population-based studies have reported improved overall survival after BCS with RT over Mx without RT.^7,8,9,10^ Mastectomy has subsequently been questioned as an equally valid surgical alternative. However, there are important confounders that may have biased these results.

When deciding on 1 of the basic 2 surgical options (BCS vs Mx), many interacting contributory factors are taken into consideration: anticipated resection volume and its association with breast volume, the tumor location in the breast and the feasibility of postoperative RT, as well as patient comorbidities, age, preferences, and beliefs.

The consequences of BCS vs Mx differ as measured by complication rates, length of hospital stay, rehabilitation time, patient-reported symptoms,^11^ body image, and quality of life.^12^ Commonly, Mx is proposed as a means to avoid postoperative RT and may therefore be more prevalent in rural areas where patients need to travel further to receive RT. However, it is important to consider that RT is not only indicated after BCS: in Sweden, postmastectomy RT is recommended for T3 tumors and extensive tumor multifocality as well as in node-positive disease, with few exceptions.

While the previously mentioned studies deliver evidence encouraging the use of BCS, it remains unclear why such survival differences would exist.^7,8,9,10^ Theories of a negative effect of larger surgery on recurrence rates and survival through the systemic release of growth factors and inflammatory effects have not been sufficiently corroborated, so Mx in itself may not be an independent factor for worse survival.^13^ Selection mechanisms and unmeasured confounders must be suspected. For example, BCS is less common in women with a lower socioeconomic status,^14^ which in turn is associated with multimorbidity,^15^ a more advanced stage at presentation,^16^ lower rates of adjuvant chemotherapy,^17^ and worse survival.^18,19,20^ Furthermore, comorbidity is associated with choice of systemic and locoregional treatment^21,22,23^ and survival.^21,24^

To further dissect the association of locoregional treatment with survival, this large population-based cohort study investigates the association of socioeconomic factors and comorbidity with overall and breast cancer–specific survival after BCS with RT, Mx with, and Mx without postoperative RT.

## Methods

This cohort study used prospectively collected data from the Swedish National Breast Cancer Register (NKBC), with national coverage since 1992 and harmonized online reporting since 2008. The NKBC includes date of diagnosis, age, sex, invasiveness, primary tumor and lymph node characteristics, metastases, date and type of surgery, oncological treatment, and follow-up. The register is 98% to 99% complete, and a 2019 validation showed a greater than 90% overlap between NKBC and validation data.^25^

From the NKBC, we included all patients diagnosed as having primary invasive breast cancer from January 1, 2008, until December 31, 2017, who underwent breast surgery with known surgery date, known tumor size of up to 50 mm (T1-2), no more than 10 positive lymph nodes (N0-2), and available data on planned or given adjuvant RT. These inclusion criteria were chosen to select patients in whom choice of locoregional treatment may have an independent survival effect and who likely would have had a choice between BCS and Mx. For women with bilateral breast cancer, we selected the side with the larger tumor and/or more nodal metastases. The cohort was individually linked to the Swedish National Patient Registers including inpatient and outpatient care, the Cause of Death Register at the National Board of Health and Welfare, and population registers at Statistics Sweden holding demographic and socioeconomic information, using the personal identification number assigned to all Swedish residents. One woman with a registered death date prior to diagnosis was excluded, as were 46 women with reused personal identification numbers.

### Locoregional Treatment

Locoregional treatment was categorized as BCS with RT (BCS+RT), Mx with RT (Mx+RT), or Mx without RT (Mx-RT). The use of reconstructive or oncoplastic procedures was not considered. Because the omission of whole-breast irradiation after BCS was not in accordance with Swedish guidelines, 2390 women treated with BCS but not receiving adjuvant RT were excluded, leaving 48 986 women for the final analysis (eFigure 1 in the Supplement).

Radiotherapy target and dose were not sufficiently available, and adjuvant RT was therefore treated as a binary variable (yes/no). According to Swedish guidelines for the relevant years, RT to regional lymph nodes was recommended in case of regional macrometastatic disease independent of the type of axillary surgery performed. In case of neoadjuvant chemotherapy, clinical node positivity or a macrometastasis on pretreatment sentinel node biopsy was an indication for regional RT, regardless of the type and results of postchemotherapy axillary staging. After neoadjuvant chemotherapy, any size of axillary metastasis was an indication for regional RT. Micrometastatic axillary disease was no indication for adjuvant regional RT, and in case of 1 single macrometastasis in a low-grade tumor and BCS, regional RT could be omitted.

Locoregional treatment not following national guidelines (ie, no RT after mastectomy despite nodal involvement) occurred in 2542 women (5.2%). Potential overtreatment with RT after mastectomy in T1N0 and T2N0 (1701 women, 3.5%) was possibly owing to extensive multifocality because only the largest tumor focus was registered.

### Tumor Characteristics and Treatment

In the NKBC, clinical pretreatment tumor size and nodal status are registered in accordance with the TNM classification (cN; cT), while exact invasive tumor size (in millimeters), number, and size of nodal metastases are registered postoperatively. Status variables unaffected by treatment were selected, ie, pretreatment clinical variables in case of neoadjuvant treatment (cT; cN) and histopathological variables in case of primary surgery (pT; pN). Likewise, data on tumor biology (estrogen receptor [ER] and progesterone receptor [PR] status, Erb-B2 receptor tyrosine kinase 2 [*ERBB2*] amplification, and proliferation) were based on the pretreatment core biopsy and on the tumor specimen, respectively. Tumor size (T) was categorized into T1 and T2, and lymph node status into N0, N1, and N2 in accordance with the eighth edition of the *AJCC Cancer Staging Manual*.^26^ Both variables were then combined into prognostic groups (T1N0, T1N1, T1N2, T2N0, T2N1, and T2N2). Estrogen receptor and PR were considered negative if less than 10%. The *ERBB2 *amplification was confirmed by an immunohistochemistry (IHC) score of 3+ or by in situ hybridization, performed in case of score of 2+. Hormone receptor–positive (HR+) tumors were ER+ and/or PR+, and hormone receptor negative (HR−) tumors were ER− and PR−. Subtypes were classified as HR+*ERBB2*−, HR+*ERBB2*+, HR−*ERBB2*+, and HR−*ERBB2*−. Oncological treatment included RT, chemotherapy (CT) (yes/no), endocrine treatment (yes/no), and targeted therapy (yes/no).

### Comorbidities

From the National Patient Registers, both main and contributing diagnoses of any comorbidity between 2008 and 2017 and within 12 months before treatment, listed in the Royal College of Surgeons Charlson Comorbidity Index (CCI; eTable 1 in the Supplement), were extracted.^27^ For patients with breast cancer diagnosed in 2008, comorbidities registered in 2008 were used.

### Education, Income, and Country of Birth

From the Longitudinal Integrated Database for Health Insurance and Labour Market Studies (LISA) database at Statistics Sweden, information on education and income for 2008 to 2017 was individually linked. The highest attained education by the year preceding the diagnosis of breast cancer was categorized as 9 years or less (primary), 10 to 13 years (secondary), and more than 13 years (tertiary). Family income in the calendar year prior to cancer surgery was used to reflect socioeconomic status and categorized into quartiles (low [Q1: 0 to 25%], middle [Q2-Q3: 25% to 75%], and high [Q4: 75% to 100%]). Income was adjusted for inflation over the study period. Country of birth was categorized as Sweden, Europe except Sweden, and any other countries. For women diagnosed in 2008, education level and income were based on data from 2008.

### Follow-up for Death

Date and cause of death was obtained from the Cause of Death Register and complemented with information from the Total Population Register at Statistics Sweden if dates were incomplete. Death owing to breast cancer was defined as death with *International Statistical Classification of Diseases and Related Health Problems, Tenth Revision (ICD-10) *code C50 as the registered cause of death.

### Ethical Considerations

The study was approved by the regional Ethical Review Authority in Stockholm (2017/2493-31), under the explicit condition that no specific informed consent was obtained other than the general consent for the use of personal data when accepting registration in NKBC. Registration in the remaining national registers is mandatory by law without consent.

### Statistical Methods

Start of follow-up was from date of surgery until end of follow-up at death or end of study in September 2019. We assessed death owing to any cause (overall survival [OS]) and death owing to breast cancer (breast cancer–specific survival [BCSS]), for which deaths owing to other causes than breast cancer were censored. Unadjusted survival proportions were estimated using the Kaplan-Meier method and compared using the log-rank test. Overall and breast cancer–specific mortality rates were modeled and adjusted using Cox regression, with time since surgery as the underlying timescale. Associations between locoregional treatment (BCS+RT, Mx-RT, and Mx+RT) and mortality rates are reported as hazard ratios (HRs) with 95% CIs. First, models were stepwise adjusted for confounders (age, year, region, prognostic group, Nottingham grade, subtype, socioeconomic factors, and CCI). The adjustment for grade and subtype was by stratification with separate baseline hazards, thereby accounting for nonproportional hazards in these variables. Second, we estimated HRs for locoregional treatment by prognostic group. In a final step, HRs for locoregional treatment were estimated for short (0-5 years) and long (>5 years) follow-up separately. In the final model, all variables except CCI fulfilled the proportional hazard assumption. Hence, we assessed a model with a time-varying effect of CCI as a sensitivity analysis, the results of the exposure variable of interest (locoregional treatment); however, this remained unchanged to the second decimal. Thus, the final model used in the main analysis did not include time-varying effects in CCI. Women with missing information on any covariates in the models were excluded. The significance level was .05 and all tests were 2-sided. All statistical analyses were performed using R, version 4.0.1 (R Foundation).

## Results

Among 48 986 women, 29 367 (59.9%) had received BCS+RT, 12 413 (25.3%) received Mx-RT, and 7206 (14.7%) received Mx+RT; Table 1. Median follow-up was 6.28 years (range, 0.01-11.70 years). Women in the BCS+RT group were more often within the age span of Swedish mammography screening (40-74 years) and had smaller tumors with less nodal involvement than women in the Mx+RT group. Women in the Mx-RT group had the highest mean age and similar rates of nodal involvement as the BCS+RT group. Neoadjuvant treatment, adjuvant chemotherapy, and targeted treatment were most common in the Mx+RT group where the proportion of larger tumors (T2) and of nodal involvement (both N1 and N2) was largest, which was mirrored in the distribution over the prognostic groups. There were fewer HR+/*ERBB2*− and more high-grade tumors in the Mx+RT group compared with BCS+RT. Women with Mx-RT had lower education levels and a lower family income, while women in both Mx groups had more comorbidities than those with BCS+RT. The distribution of locoregional treatments varied across Swedish regions but not over calendar periods.

**Table 1.** Patient, Tumor, and Treatment Characteristics by Locoregional Treatment

Characteristic	No. (%)				*P* value
	BCS+RT (n = 29 367)	Mx-RT (n = 12 413)	Mx+RT (n = 7206)	Total (n = 48 986)	
Follow-up time from surgery, median (range), y	6.35 (0.02-11.70)	6.20 (0.01-11.69)	6.02 (0.12-11.66)	6.28 (0.01-11.70)	<.001
No. of deaths					
OS	2272 (7.7)	2912 (23.5)	1389 (19.3)	6573 (13.4)	<.001
BCSS	727 (2.5)	771 (6.2)	815 (11.3)	2313 (4.7)	<.001
Age at diagnosis, y					
<40	736 (2.5)	451 (3.6)	643 (8.9)	1830 (3.7)	<.001
40-49	4170 (14.2)	1457 (11.7)	1503 (20.9)	7130 (14.6)	
50-64	12 424 (42.3)	3212 (25.9)	2366 (32.8)	18 002 (36.7)	
65-74	10 134 (34.5)	3383 (27.3)	1629 (22.6)	15 146 (30.9)	
≥75	1903 (6.5)	3910 (31.5)	1065 (14.8)	6878 (14.0)	
Mean (SD)	60.8 (10.5)	66.3 (14.2)	58.7 (13.9)	61.9 (12.4)	<.001
Median (range)	62 (22-94)	68 (21-97)	59 (19-95)	63 (19-97)	<.001
Year of surgery					
2008-2009	5044 (17.2)	2665 (21.5)	1404 (19.5)	9113 (18.6)	<.001
2010-2011	6076 (20.7)	2934 (23.6)	1636 (22.7)	10 646 (21.7)	
2012-2013	6759 (23.0)	2782 (22.4)	1571 (21.8)	11 112 (22.7)	
2014-2015	7437 (25.3)	2683 (21.6)	1691 (23.5)	11 811 (24.1)	
2016-2017	4051 (13.8)	1349 (10.9)	904 (12.5)	6304 (12.9)	
T stage^a^					
T1	23 266 (79.2)	7114 (57.3)	2643 (36.7)	33 023 (67.4)	<.001
T2	6101 (20.8)	5299 (42.7)	4563 (63.3)	15 963 (32.6)	
T stage^b^					
T1mi	177 (0.6)	116 (0.9)	43 (0.6)	336 (0.7)	<.001
T1a	1146 (3.9)	435 (3.5)	97 (1.3)	1678 (3.4)	
T1b	7082 (24.1)	1479 (11.9)	379 (5.3)	8940 (18.3)	
T1c	14 737 (50.2)	5035 (40.6)	1935 (26.9)	21 707 (44.3)	
T1	124 (0.4)	49 (0.4)	189 (2.6)	362 (0.7)	
T2	6101 (20.8)	5299 (42.7)	4563 (63.3)	15 963 (32.6)	
Lymph node status					
N0	22 933 (78.1)	9871 (79.5)	1701 (23.6)	34 505 (70.4)	<.001
N1	5666 (19.3)	2268 (18.3)	3989 (55.4)	11 923 (24.3)	
N2	768 (2.6)	274 (2.2)	1516 (21.0)	2558 (5.2)	
Prognostic groups					
T1N0	19 147 (65.2)	6021 (48.5)	805 (11.2)	25 973 (53.0)	<.001
T1N1	3732 (12.7)	1036 (8.3)	1420 (19.7)	6188 (12.6)	
T1N2	387 (1.3)	57 (0.5)	418 (5.8)	862 (1.8)	
T2N0	3786 (12.9)	3850 (31.0)	896 (12.4)	8532 (17.4)	
T2N1	1934 (6.6)	1232 (9.9)	2569 (35.7)	5735 (11.7)	
T2N2	381 (1.3)	217 (1.7)	1098 (15.2)	1696 (3.5)	
Primary treatment					
Surgery	28 859 (98.3)	12 149 (97.9)	6038 (83.8)	47 046 (96.0)	<.001
Neoadjuvant systemic treatment	508 (1.7)	264 (2.1)	1168 (16.2)	1940 (4.0)	
Bilateral breast cancer					
No	28 761 (97.9)	11 965 (96.4)	7034 (97.6)	47 760 (97.5)	<.001
Yes	606 (2.1)	448 (3.6)	172 (2.4)	1226 (2.5)	
Histological invasive subtype					
Ductal	23 607 (80.4)	9367 (75.5)	4920 (68.3)	37 894 (77.4)	<.001
Lobular	3052 (10.4)	1906 (15.4)	935 (13.0)	5893 (12.0)	
Other	2114 (7.2)	848 (6.8)	208 (2.9)	3170 (6.5)	
Missing	594 (2.0)	292 (2.4)	1143 (15.9)	2029 (4.1)	
Nottingham grade					
1	7388 (25.2)	2086 (16.8)	514 (7.1)	9988 (20.4)	<.001
2	14 346 (48.9)	6296 (50.7)	2878 (39.9)	23 520 (48.0)	
3	6873 (23.4)	3602 (29.0)	2596 (36.0)	13071 (26.7)	
Missing	760 (2.6)	429 (3.5)	1218 (16.9)	2407 (4.9)	
*ERBB2* amplification					
Yes	2932 (10.0)	1619 (13.0)	1425 (19.8)	5976 (12.2)	<.001
No	25 304 (86.2)	9979 (80.4)	5454 (75.7)	40 737 (83.2)	
Missing	1131 (3.9)	815 (6.6)	327 (4.5)	2273 (4.6)	
ER status					
Positive	23 512 (80.1)	9091 (73.2)	5066 (70.3)	37 669 (76.9)	<.001
Negative	2197 (7.5)	1091 (8.8)	933 (12.9)	4221 (8.6)	
Missing	3658 (12.5)	2231 (18.0)	1207 (16.7)	7096 (14.5)	
PR status					
Positive	19 541 (66.5)	7185 (57.9)	3982 (55.3)	30 708 (62.7)	<.001
Negative	5529 (18.8)	2622 (21.1)	1849 (25.7)	10 000 (20.4)	
Missing	4297 (14.6)	2606 (21.0)	1375 (19.1)	8278 (16.9)	
Subtype					
HR+*ERBB2*-	21 177 (72.1)	7852 (63.3)	4193 (58.2)	33 222 (67.8)	<.001
HR+*ERBB2*+	1960 (6.7)	919 (7.4)	786 (10.9)	3665 (7.5)	
HR-*ERBB2*+	519 (1.8)	346 (2.8)	356 (4.9)	1221 (2.5)	
HR-*ERBB2*-	1536 (5.2)	678 (5.5)	519 (7.2)	2733 (5.6)	
Missing	4175 (14.2)	2618 (21.1)	1352 (18.8)	8145 (16.6)	
Chemotherapy					
Yes	8168 (27.8)	2727 (22.0)	4377 (60.7)	15 272 (31.2)	<.001
No	21 199 (72.2)	9686 (78.0)	2829 (39.3)	33 714 (68.8)	
Endocrine treatment					
Yes	18 932 (64.5)	8004 (64.5)	4794 (66.5)	31 730 (64.8)	.003
No	10 435 (35.5)	4409 (35.5)	2412 (33.5)	17 256 (35.2)	
Targeted treatment					
Yes	2174 (7.4)	960 (7.7)	1164 (16.2)	4298 (8.8)	<.001
No	27 193 (92.6)	11 453 (92.3)	6042 (83.8)	44 688 (91.2)	
Country of birth					
Sweden	25 208 (85.8)	10 797 (87.0)	6051 (84.0)	42 056 (85.9)	<.001
Europe, not Sweden	2791 (9.5)	1206 (9.7)	717 (10.0)	4714 (9.6)	
Asia, Africa, North/South America, Australia, and Oceania	1354 (4.6)	401 (3.2)	431 (6.0)	2186 (4.5)	
Missing	14	9 (0.1)	7 (0.1)	30 (0.1)	
Region of residence					
Stockholm/Gotland	7377 (25.1)	1997 (16.1)	1770 (24.6)	11 144 (22.7)	<.001
Uppsala/Örebro	6222 (21.2)	2313 (18.6)	1743 (24.2)	10 278 (21.0)	
Southeast	2631 (9.0)	1437 (11.6)	1006 (14.0)	5074 (10.4)	
South	5323 (18.1)	2889 (23.3)	1226 (17.0)	9438 (19.3)	
West	4978 (17.0)	2769 (22.3)	939 (13.0)	8686 (17.7)	
North	2836 (9.7)	1008 (8.1)	522 (7.2)	4366 (8.9)	
Family income					
Low	5527 (18.8)	4282 (34.5)	1609 (22.3)	11 418 (23.3)	<.001
Middle	15 580 (53.1)	5720 (46.1)	3524 (48.9)	24 824 (50.7)	
High	8211 (28.0)	2399 (19.3)	2063 (28.6)	12 673 (25.9)	
Missing	49 (0.2)	12 (0.1)	10 (0.1)	71 (0.1)	
Highest attained education					
≤9 y (Primary)	5596 (19.1)	3748 (30.2)	1447 (20.1)	10 791 (22.0)	<.001
10-13 y (Secondary)	12 867 (43.8)	4819 (38.8)	2962 (41.1)	20 648 (42.2)	
>13 y (Tertiary)	10 664 (36.3)	3680 (29.6)	2707 (37.6)	17 051 (34.8)	
Missing	240 (0.8)	166 (1.3)	90 (1.2)	496 (1.0)	
Charlson comorbidity index within 1 y before treatment					
Mean (SD)	0.268 (1.12)	0.525 (1.53)	1.05 (2.27)	0.449 (1.47)	<.001
Median (range)	0 (0-12.0)	0 (0-14.0)	0 (0-10.0)	0 (0-14.0)	<.001
Charlson Comorbidity Index within 1 y before treatment					
0	26 810 (91.3)	10 209 (82.2)	5664 (78.6)	42 683 (87.1)	<.001
≥1	2557 (8.7)	2204 (17.8)	1542 (21.4)	6303 (12.9)	

Abbreviations: BCS, breast-conserving surgery; BCSS, breast cancer–specific survival; *ERBB2*, Erb-B2 receptor tyrosine kinase 2; ER, estrogen receptor; HR, hormone receptor status; Mx, mastectomy; OS, overall survival; PR, progesterone receptor; RT, radiotherapy.

^a^ Histopathologic tumor size for primarily operated patients but clinical T stage for patients receiving neoadjuvant treatment.

^b^ Histopathologic tumor size available only for primarily operated-on patients.

**Figure 1.**
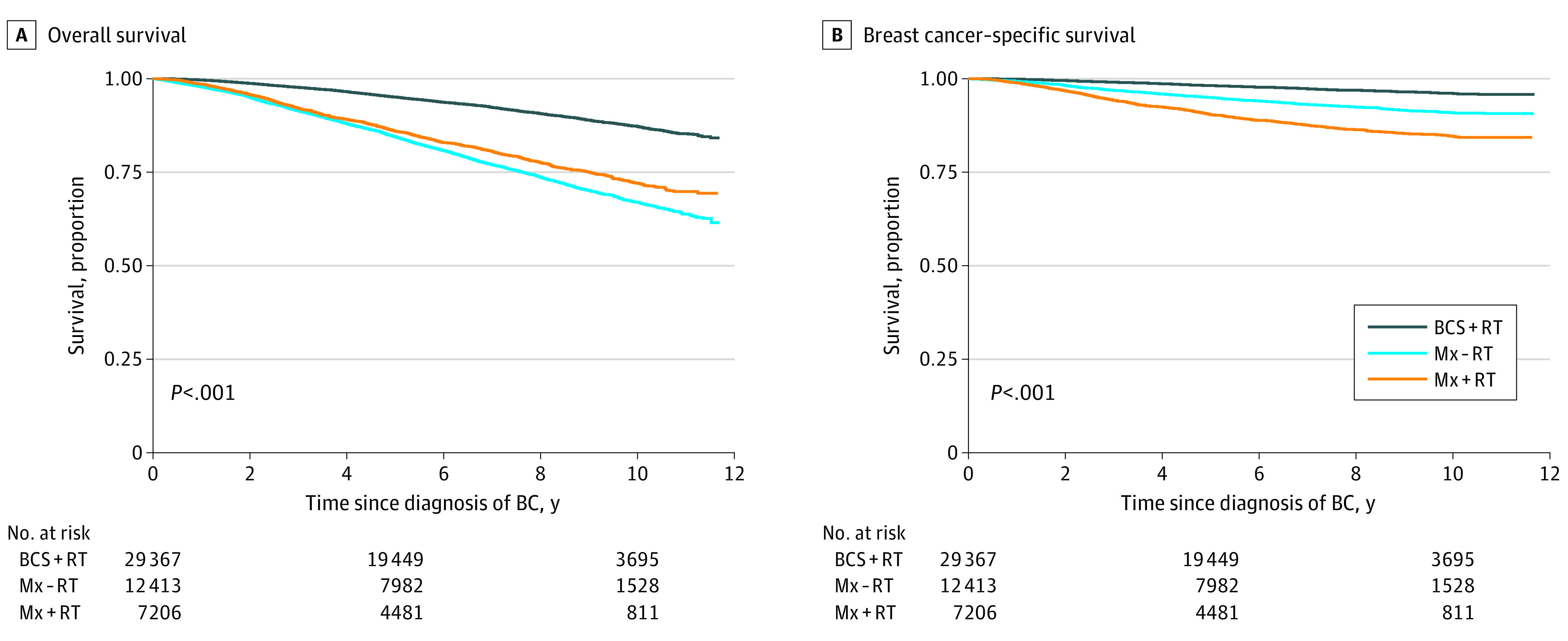
Survival Proportions by Locoregional Treatment Group for Overall Survival (A) and Breast Cancer–Specific Survival (B) BC indicates breast cancer; BCS, breast-conserving surgery; Mx, mastectomy; RT, radiotherapy.

**Figure 2.**
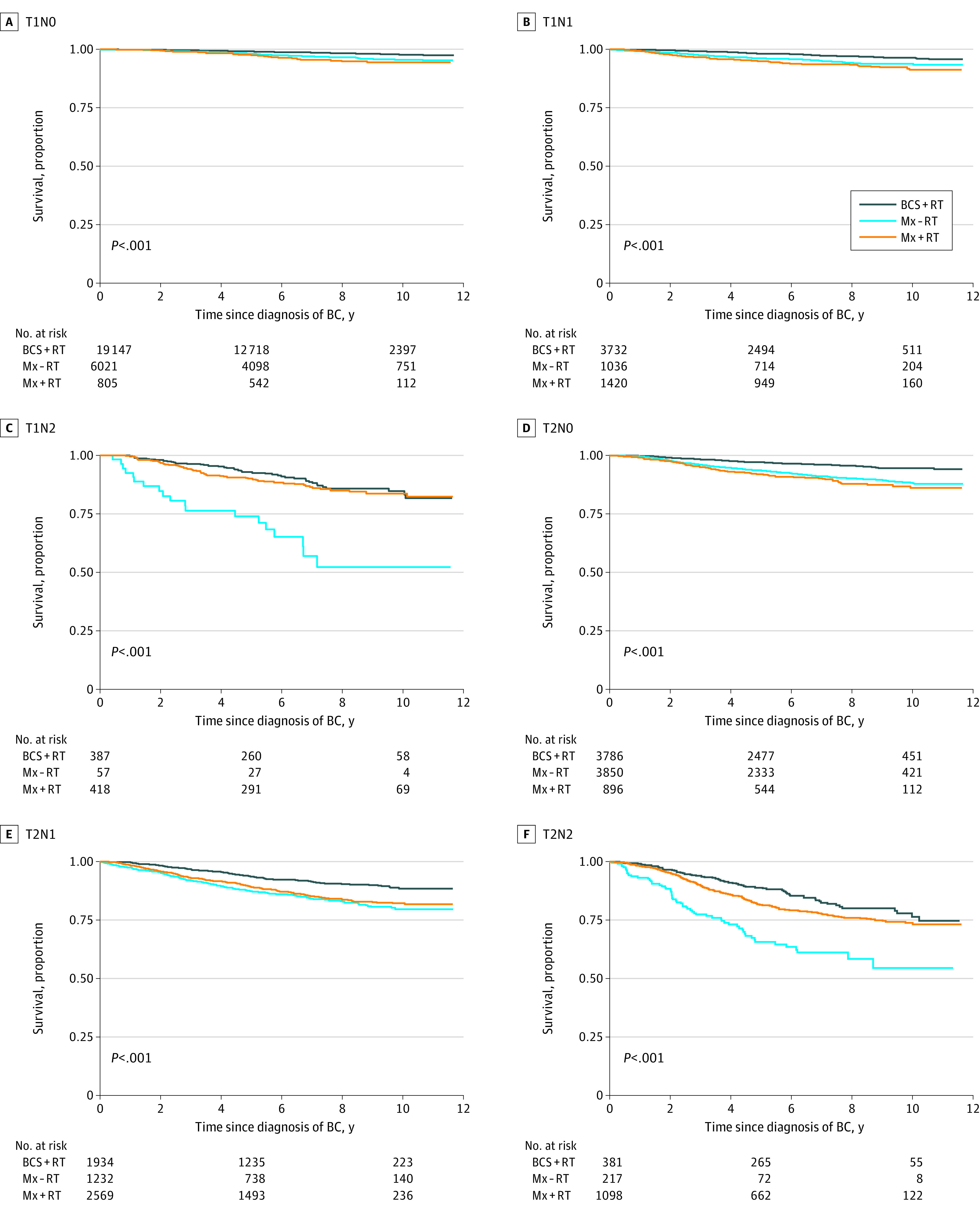
Survival Proportions for Breast Cancer-Specific Survival by Locoregional Treatment Group and Prognostic Group BC indicates breast cancer; BCS, breast-conserving surgery; Mx, mastectomy; RT, radiotherapy.

In total, 6573 deaths occurred during follow-up, of which 2313 (35.2%) were owing to breast cancer. Five-year survival was 91.1% (OS) and 96.3% (BCSS), while 10-year survival was 79.5% (OS) and 93.1% (BCSS). In the unadjusted analysis, Mx-RT was associated with the lowest OS (Figure 1A) and Mx+RT with the lowest BCSS (Figure 1B). The corresponding 5-year and 10-year survival proportions by locoregional treatment groups are presented in Table 2. When stratified by prognostic group, Mx-RT was particularly associated with lower BCSS among prognostic groups with a clear indication for adjuvant RT, ie, T1N2 and T2N2 (Figure 2). Mastectomy with RT was associated with a lower BCSS than BCS+RT across all prognostic groups (Figure 2), and with the lowest BCSS in all age groups (eFigure 2 in the Supplement). For OS, Mx-RT was associated with the lowest survival in all prognostic groups (eFigure 3 in the Supplement), and Mx+RT with the lowest survival across all age groups (eFigure 4 in the Supplement).

**Table 2.** Hazard Ratios of OS and BCSS for Locoregional Treatment Adjusted Stepwise for Tumor Characteristics, Treatment, Socioeconomic Status, and Charlson Comorbidity Index

Variable	**Survival % (95% CI)**		**HR (95% CI)**			
	5-y	10-y	**Model 1**^a^	**Model 2**^b^	**Model 3**^c^	**Model 4**^d^
OS						
BCS+RT	95.1 (94.9-95.4)	87.3 (86.7-87.9)	1 [Reference]	1 [Reference]	1 [Reference]	1 [Reference]
Mx-RT	84.5 (83.9-85.2)	67.0 (65.9-68.2)	1.94 (1.82-2.06)	1.91 (1.78-2.06)	1.83 (1.70-1.97)	1.79 (1.66-1.92)
Mx+RT	86.0 (85.2-86.9)	72.1 (70.7-73.7)	2.36 (2.21-2.53)	1.24 (1.13-1.37)	1.25 (1.13-1.38)	1.24 (1.13-1.37)
BCSS						
BCS+RT	98.2 (98.0-98.3)	96.1 (95.8-96.5)	1 [Reference]	1 [Reference]	1 [Reference]	1 [Reference]
Mx-RT	95.0 (94.6-95.4)	91.0 (90.3-91.7)	1.89 (1.69-2.10)	1.71 (1.50-1.96)	1.67 (1.46-1.91)	1.66 (1.45-1.90)
Mx+RT	90.5 (89.7-91.2)	84.6 (83.5-85.8)	4.30 (3.88-4.76)	1.25 (1.08-1.45)	1.25 (1.08-1.46)	1.26 (1.08-1.46)

Abbreviations: BCS, breast-conserving surgery; BCSS, breast cancer–specific survival; Mx, mastectomy; OS, overall survival; RT, radiotherapy.

^a^ Adjusted for age, calendar year, and region of residence at diagnosis.

^b^ Adjusted for same variables as model 1 plus Nottingham grade, prognostic group, and subtype.

^c^ Adjusted for same variables as model 2 plus education, family income, and country of birth.

^d^ Adjusted for same variables as model 3 plus Charlson Comorbidity Index one year prior to operation.

In age-, year-, and region-adjusted Cox regression models, Mx-RT was associated with an increased overall mortality rate (HR, 1.94; 95% CI, 1.82-2.06) and breast-cancer-specific mortality rate (HR, 1.89; 95% CI, 1.69-2.10) (Table 2, Model 1). The associations were even stronger for Mx+RT (OS: HR, 2.36; 95% CI, 2.21-2.53; BCSS: HR, 4.30; 95% CI, 3.88-4.76, model 1). After adjustment for tumor stage, subtype, and grade, the associations were reduced but remained nevertheless significant (model 2). Further adjustments for education level, family income, and country of birth (model 3) and the addition of CCI (model 4) did not alter the estimates substantially.

When stratifying by prognostic group, the associations varied substantially (Table 3). Mastectomy without RT was associated with increased overall and breast cancer–specific mortality rates compared with BCS+RT regardless of prognostic group, with the exception of T1N1 where no association was found (BCSS: HR, 0.99; 95% CI, 0.60-1.64). Mastectomy with RT was associated with an increased overall mortality rate in T1N0, T1N1, and T2N0, but not T1N2. Among T2N1 and T2N2, Mx+RT was not associated with increased overall mortality rates, although point estimates were moderately increased.

**Table 3.** Hazard Ratios of OS and BCSS for Locoregional Treatment by Prognostic Groups

Variable	OS			BCSS		
	No. (deaths)	HR (95% CI)^a^	*P* value	No. (deaths)	HR (95% CI)^a^	*P* value
Overall						
BCS+RT	29 367 (2272)	1 [Reference]	NA	29 367 (727)	1 [Reference]	NA
Mx-RT	12 413 (2912)	1.79 (1.66-1.92)	<.001	12 413 (771)	1.66 (1.45-1.91)	<.001
Mx+RT	7206 (1389)	1.24 (1.13-1.37)	<.001	7206 (815)	1.28 (1.10-1.49)	.001
T1N0						
BCS+RT	19 147 (1233)	1 [Reference]	NA	19 147 (243)	1 [Reference]	NA
Mx-RT	6021 (947)	1.56 (1.40-1.74)	<.001	6021 (166)	1.68 (1.31-2.15)	<.001
Mx+RT	805 (76)	1.37 (1.03-1.84)	.03	805 (30)	1.79 (1.08-2.99)	.03
T1N1						
BCS+RT	3732 (269)	1 [Reference]	NA	3732 (89)	1 [Reference]	NA
Mx-RT	1036 (261)	2.02 (1.61-2.53)	<.001	1036 (49)	0.99 (0.60-1.64)	.97
Mx+RT	1420 (190)	1.43 (1.13-1.81)	.003	1420 (86)	1.51 (1.02-2.25)	.04
T1N2						
BCS+RT	387 (66)	1 [Reference]	NA	387 (43)	1 [Reference]	NA
Mx-RT	57 (36)	3.79 (2.10-6.83)	<.001	57 (19)	5.05 (2.32-10.95)	<.001
Mx+RT	418 (96)	1.31 (0.88-1.96)	.18	418 (56)	1.29 (0.77-2.15)	.34
T2N0						
BCS+RT	3786 (372)	1 [Reference]	NA	3786 (140)	1 [Reference]	NA
Mx-RT	3850 (1021)	1.70 (1.46-1.98)	<.001	3850 (299)	1.66 (1.29-2.13)	<.001
Mx+RT	896 (140)	1.53 (1.17-2.00)	.002	896 (85)	1.64 (1.07-2.51)	.02
T2N1						
BCS+RT	1934 (244)	1 [Reference]	NA	1934 (149)	1 [Reference]	NA
Mx-RT	1232 (494)	2.08 (1.70-2.55)	<.001	1232 (175)	1.53 (1.13-2.08)	.006
Mx+RT	2569 (519)	1.32 (1.08-1.60)	.007	2569 (328)	1.21 (0.93-1.59)	.16
T2N2						
BCS+RT	381 (88)	1 [Reference]	NA	381 (63)	1 [Reference]	NA
Mx-RT	217 (153)	2.77 (1.92-3.98)	<.001	217 (63)	2.37 (1.47-3.82)	<.001
Mx+RT	1098 (368)	1.29 (0.95-1.74)	.10	1098 (230)	1.26 (0.87-1.82)	.23

Abbreviations: BCS, breast-conserving surgery; BCSS, breast cancer–specific survival; HR, hazard ratio; Mx, mastectomy; OS, overall survival; RT, radiotherapy.

^a^ Adjusted for age and calendar year at diagnosis, region, country of birth, family income, highest education, Nottingham grade, hormone receptor status (estrogen receptor/progesterone receptor), *ERBB2*, and Charlson Comorbidity Index 1 year prior to surgery.

When stratifying by follow-up time, the adjusted associations for all prognostic groups combined did not vary by short (0-5 years) or long (>5 years) follow-up for overall mortality rates, whereas the associations for breast cancer–specific mortality rates were stronger shortly (0-5 years) after surgery (eTable 2 in the Supplement). These association patterns were similar when stratified by prognostic groups, but the pattern was less consistent for breast cancer–specific mortality, where some prognostic groups had stronger associations with surgical treatment in the longer follow-up (Mx+RT: T1N0, T2N0).

Of special clinical interest are women who would probably have the choice of 2 guideline-adherent locoregional treatment alternatives: women with T1-2N0 tumors are commonly suitable for BCS+RT or Mx-RT, and women with T1-2N1-2 tumors are often suitable for BCS+RT or Mx+RT. In T1-2N0, adjusted HRs for both OS and BCSS showed a significant benefit of BCS+RT over Mx-RT. Among women with T1-2N1-2, those with T1N1 had lower mortality rates (OS and BCSS) with BCS+RT than with Mx+RT, and those with T2N1 had a lower mortality rate for OS only. For the remaining groups, no significant associations were found (Table 3).

## Discussion

The findings of this report confirm the superiority of BCS with RT over Mx with an overall and breast cancer–specific relative survival gain of 56% to 70% in node-negative patients. This association resisted adjustment for tumor biology and status, socioeconomic background, and comorbidities. The same association was observed in lower-burden, node-positive disease, but not in women with higher nodal stage. Because there was no inferior survival for BCS in node-positive patients, this report gives no support to advocate Mx in women without specific risk factors, such as a strong family history or gene mutations.

There are complex interactions between breast cancer survival, socioeconomic status, and comorbidity. Individuals with a lower socioeconomic status present with more advanced disease, have a lower adherence to mammography screening, are less likely to receive chemotherapy, and have inferior survival rates.^16,17,18^ In addition, lifestyle factors increasing cancer risk and impacting survival, such as obesity and smoking, are more common in socioeconomically deprived groups, in addition to comorbidities that negatively affect completion rates of systemic therapy.^28^ Comorbidity is a mediator of death and will thus be associated with OS but may also be associated with BCSS by modulating adjuvant treatments. While a significant association of these factors with survival differences between BCS and Mx was anticipated, the adjustment for socioeconomic background and comorbidity was not associated with HRs for OS or BCSS. It is unlikely to assume that BCS would have some intrinsic positive association with survival, although it provides better health-related quality of life and was associated with fewer postoperative complications than Mx.^29^ Although tangential whole-breast irradiation after BCS reduces the risk of axillary recurrence in patients with node-negative disease,^30^ no independent association of RT could be observed in this study. Thus, further unmeasured confounding must be suspected: first, no Swedish register provides information on smoking or body mass index, and second, the CCI lacks important potential contributory diagnoses, such as alcohol and drug abuse as well as psychiatric disorders. Furthermore, there may be complex and synergistic interactions between multiple confounders that are difficult to control for in an observational setting.

The decision for BCS vs Mx is multifaceted. Importantly, it is influenced by the degree of patient-perceived information and involvement, fear of cancer recurrence, the perception that health outweighs breast retention, and the risk of reoperation in case of positive margins.^14,31^ These obstacles can be overcome by dedicated patient information and education, and a collaborative weighing of pros and cons by the treating clinician and the patient. It is striking that extensive breast surgery is more prevalent in node-positive disease despite suitability for breast conservation, indicating a misconception of safety, probably both from a patient and a physician perspective. In short, more extensive breast surgery does not appear to save any lives.

### Limitations

The strength of this work is the population-based setting, providing a representative sample with complete follow-up and detailed clinical data. To our knowledge, this is the first report that integrates socioeconomic status and comorbidity in survival analyses juxtaposing locoregional treatments. Limitations include the lack of potential confounders, such as smoking and body mass index, and the potential underestimation of unlisted comorbid conditions. However, in terms of capturing comorbidities, both hospital and outpatient data and both main and contributing diagnoses were included, assuring a sound validity of CCI. Follow-up is still short considering late recurrences, especially in luminal-type breast cancer. Therefore, it will be important to replicate this survival analysis in additional studies.

## Conclusions

In conclusion, this report adds evidence to support the recommended use of BCS with RT in both node-negative and node-positive breast cancer. Neither socioeconomic background and comorbidity nor the addition of postoperative RT after mastectomy diminished survival differences. This report casts additional doubt on the practice to offer mastectomy to patients who are suitable candidates for breast conservation.

## Notes

Supplement. **eTable 1.** ICD-10 codes based on the Royal College of Surgeons’ adaptation of the Charlson Comorbidity Index **eTable 2.** Hazard ratios of OS and BCSS for locoregional treatment and prognostic group by short (0-5 years) and long (>5 years) follow-up **eFigure 1.** CONSORT diagram for all primary breast cancer cases diagnosed in Sweden 2008-2017 **eFigure 2.** Survival proportions for breast cancer-specific survival by locoregional treatment group and age **eFigure 3.** Survival proportions for overall survival by locoregional treatment group and prognostic group **eFigure 4.** Survival proportions for overall survival by locoregional treatment group and age
