# Supplementary material for: Survival After Breast Conservation vs Mastectomy Adjusted for Comorbidity and Socioeconomic Status: A Swedish National 6-Year Follow-up of 48 986 Women
Source: JAMA Surg. 2021 May 5;156(7):628–37. doi: 10.1001/jamasurg.2021.1438 (PMC8100916; doi:10.1001/jamasurg.2021.1438)
Supplement: Supplement. — eTable 1. ICD-10 codes based on the Royal College of Surgeons’ adaptation of the Charlson Comorbidity Index eTable 2. Hazard ratios of OS and BCSS for locoregional treatment and prognostic group by short (0-5 years) and long (>5 years) follow-up eFigure 1. CONSORT diagram for all primary breast cancer cases diagnosed in Sweden 2008-2017 eFigure 2. Survival proportions for breast cancer-specific survival by locoregional treatment group and age eFigure 3. Survival proportions for overall survival by locoregional treatment group and prognostic group eFigure 4. Survival proportions for overall survival by locoregional treatment group and age [file jamasurg-e211438-s001.pdf]

# Supplemental Online Content

de Boniface J, Szulkin R, Johansson ALV. Survival after breast conservation vs mastectomy adjusted for comorbidity and socioeconomic status: a Swedish national 6-year follow-up of 48 986 women. *JAMA Surg*. Published online May 5, 2021. doi:10.1001/jamasurg.2021.1438

**eTable 1.** ICD-10 codes based on the Royal College of Surgeons' adaptation of the Charlson Comorbidity Index

**eTable 2.** Hazard ratios of OS and BCSS for locoregional treatment and prognostic group by short (0-5 years) and long (>5 years) follow-up

**eFigure 1.** CONSORT diagram for all primary breast cancer cases diagnosed in Sweden 2008-2017

**eFigure 2.** Survival proportions for breast cancer-specific survival by locoregional treatment group and age

**eFigure 3.** Survival proportions for overall survival by locoregional treatment group and prognostic group

**eFigure 4.** Survival proportions for overall survival by locoregional treatment group and age

This supplemental material has been provided by the authors to give readers additional information about their work.

© 2021 de Boniface J et al. *JAMA Surgery.*

## eTable 1. ICD-10 codes based on the Royal College of Surgeons' adaptation of the Charlson Comorbidity Index

| Disease category | Weight | ICD-10 |
|---|---|---|
| Diabetes mellitus | 1 | E10, E11, E12, E13, E14 |
| Dementia | 1 | F00, F01, F02, F03, G30, G31, A81, F05 |
| Cerebrovascular Disease | 1 | G45, G46, I60, I61, I62, I63, I64, I65, I66, I67, I68, I69 |
| Congestive Heart Failure | 1 | I11, I42, I43, I50, I51 |
| Congestive Heart Failure or Renal Disease * | 1 | I13 |
| Myocardial Infarction | 1 | I21, I22, I23 |
| Congestive Heart Failure or Myocardial Infarction * | 1 | I25 |
| Chronic Pulmonary Disease | 1 | I26, I27, J40, J41, J42, J43, J44, J45, J46, J47, J60, J61, J62, J63, J64, J65, J66, J67, J68, J70 |
| Peripheral Vascular Disease | 1 | I70, I71, I72, I73, I77, K55, Z95 |
| Rheumatological Disease | 1 | M05, M06, M09, M32, M33, M34, M35, M36, M12, M31 |
| Any malignancy | 2 | C00, C01, C02, C03, C04, C05, C06, C07, C08, C09, C10, C11, C12, C13, C14, C15, C16, C17, C18, C19, C20, C21, C22, C23, C24, C25, C26, C30, C31, C32, C33, C34, C37, C38, C39, C40, C41, C43, C45, C46, C47, C48, C49, C50, C51, C52, C53, C54, C55, C56, C57, C58, C60, C61, C62, C63, C64, C65, C66, C67, C68, C69, C70, C71, C72, C73, C74, C75, C76, C80, C81, C82, C83, C84, C85, C88, C90, C91, C92, C93, C94, C95, C96, C97 |
| Hemiplegia or paraplegia | 2 | G81, G82, G83, G11 |
| Renal Disease | 2 | I12, N01, N03, N05, N07, N08, N18, N19, N25, Z49, N17, Z99 |
| Renal Disease or Liver Disease * | 2 | Z94 |
| Liver Disease | 3 | B18, I85, K70, K71, K76, I86, I98, K72 |
| AIDS/HIV | 6 | B20, B21, B22, B24 |
| Metastatic solid tumor | 6 | C77, C78, C79 |

* Due to ICD-10 being included as 3-digit codes in the patient register, it was not possible to separate these diseases.

© 2021 de Boniface J et al. *JAMA Surgery.*

**eTable 2. Hazard ratios of OS and BCSS for locoregional treatment and prognostic group by short (0-5 years) and long (>5 years) follow-up.**

| | OS | | OS | | BCSS | | BCSS | |
|---|---|---|---|---|---|---|---|---|
| | 0-5 year | | >5 year | | 0-5 year | | >5 year | |
| | HR (95% CI)[a] | p-value | HR (95% CI)[a] | p-value | HR (95% CI)[a] | p-value | HR (95% CI)[a] | p-value |
| **All prognostic groups** | | | | | | | | |
| BCS+RT | 1.00 (reference) | | 1.00 (reference) | | 1.00 (reference) | | 1.00 (reference) | |
| Mx-RT | 1.83 (1.67-2.00) | <0.001 | 1.72 (1.55-1.92) | <0.001 | 1.78 (1.52-2.09) | <0.001 | 1.45 (1.15-1.82) | 0.001 |
| Mx+RT | 1.25 (1.11-1.41) | <0.001 | 1.24 (1.07-1.43) | 0.004 | 1.34 (1.13-1.60) | <0.001 | 1.17 (0.91-1.49) | 0.224 |
| **Prognostic group:** | | | | | | | | |
| **T1N0** | | | | | | | | |
| BCS+RT | 1.00 (reference) | | 1.00 (reference) | | 1.00 (reference) | | 1.00 (reference) | |
| Mx-RT | 1.53 (1.33-1.76) | <0.001 | 1.59 (1.36-1.87) | <0.001 | 1.55 (1.12-2.13) | 0.008 | 1.88 (1.30-2.73) | <0.001 |
| Mx+RT | 1.37 (0.93-2.02) | 0.117 | 1.38 (0.89-2.13) | 0.146 | 1.54 (0.77-3.07) | 0.224 | 2.19 (1.04-4.60) | 0.039 |
| **T1N1** | | | | | | | | |
| BCS+RT | 1.00 (reference) | | 1.00 (reference) | | 1.00 (reference) | | 1.00 (reference) | |
| Mx-RT | 2.00 (1.51-2.65) | <0.001 | 2.04 (1.46-2.86) | <0.001 | 1.21 (0.68-2.17) | 0.514 | 0.58 (0.22-1.57) | 0.287 |
| Mx+RT | 1.31 (0.97-1.78) | 0.078 | 1.64 (1.13-2.37) | 0.009 | 1.73 (1.09-2.75) | 0.021 | 1.08 (0.50-2.35) | 0.842 |
| **T1N2** | | | | | | | | |
| BCS+RT | 1.00 (reference) | | 1.00 (reference) | | 1.00 (reference) | | 1.00 (reference) | |
| Mx-RT | 4.32 (2.22-8.42) | <0.001 | 2.51 (0.90-7.04) | 0.08 | 5.77 (2.37-14.02) | <0.001 | 4.31 (1.19-15.56) | 0.026 |
| Mx+RT | 1.30 (0.77-2.21) | 0.327 | 1.32 (0.73-2.40) | 0.358 | 1.62 (0.83-3.16) | 0.157 | 0.90 (0.40-2.04) | 0.809 |
| **T2N0** | | | | | | | | |
| BCS+RT | 1.00 (reference) | | 1.00 (reference) | | 1.00 (reference) | | 1.00 (reference) | |
| Mx-RT | 1.78 (1.47-2.16) | <0.001 | 1.58 (1.26-1.99) | <0.001 | 1.70 (1.26-2.29) | <0.001 | 1.55 (1.00-2.42) | 0.051 |
| Mx+RT | 1.35 (0.93-1.96) | 0.117 | 1.77 (1.21-2.60) | 0.003 | 1.39 (0.80-2.40) | 0.241 | 2.17 (1.11-4.24) | 0.023 |
| **T2N1** | | | | | | | | |

© 2021 de Boniface J et al. *JAMA Surgery.*

| | | | | | | | | |
|---|---|---|---|---|---|---|---|---|
| BCS+RT | 1.00 (reference) | | 1.00 (reference) | | 1.00 (reference) | | 1.00 (reference) | |
| Mx-RT | 2.09 (1.63-2.69) | <0.001 | 2.04 (1.49-2.78) | <0.001 | 1.68 (1.19-2.38) | 0.003 | 1.17 (0.67-2.05) | 0.573 |
| Mx+RT | 1.23 (0.95-1.58) | 0.11 | 1.49 (1.09-2.05) | 0.013 | 1.10 (0.80-1.51) | 0.566 | 1.59 (0.98-2.58) | 0.061 |
| | | | | | | | | |
| | **OS** | | **OS** | | **BCSS** | | **BCSS** | |
| | **0-5 year** | | **>5 year** | | **0-5 year** | | **>5 year** | |
| | **HR (95% CI)[a]** | **p-value** | **HR (95% CI)[a]** | **p-value** | **HR (95% CI)[a]** | **p-value** | **HR (95% CI)[a]** | **p-value** |
| **T2N2** | | | | | | | | |
| BCS+RT | 1.00 (reference) | | 1.00 (reference) | | 1.00 (reference) | | 1.00 (reference) | |
| Mx-RT | 3.45 (2.21-5.40) | <0.001 | 2.13 (1.16-3.92) | 0.015 | 3.12 (1.80-5.41) | <0.001 | 0.95 (0.30-2.97) | 0.931 |
| Mx+RT | 1.75 (1.17-2.59) | 0.006 | 0.77 (0.49-1.22) | 0.272 | 1.64 (1.04-2.59) | 0.035 | 0.66 (0.35-1.25) | 0.203 |

[a] Adjusted for age and calendar year at diagnosis, region, country of birth, family income, highest education, Nottingham grade, subtype, and Charlson Comorbidity Index one year prior to surgery.

OS, overall survival; BCSS, breast cancer-specific survival; BCS, breast-conserving surgery; Mx, mastectomy; RT, radiotherapy

© 2021 de Boniface J et al. *JAMA Surgery.*

## eFigure 1. CONSORT diagram for all primary breast cancer cases diagnosed in Sweden 2008-2017

**Data extraction from NKBC 2008-2017**
**N=66490 invasive breast cancers**

**Exclusions before register linkages:**

- No breast surgery performed, N=134
- Type of breast surgery not reported, N=4917
- Distant metastasis at diagnosis and/or no surgery planned, N=162
- Locally advanced tumors (T4), N=572
- Clinical (in neoadjuvant cases) or pathological (in primary surgery cases) tumour stage not reported, N=1141
- Non-invasive breast cancer reported incorrectly to the register, N=1
- No information on planned or received adjuvant radiotherapy, N=854

**N=58709 breast cancers (in 57237 women)**
**Linkage to Patient Register, Cause of Death Register, and LISA database for socioeconomic background**

- Second (contralateral) breast cancer, N=1472 (leaving 57237 women as above but reducing number of cancer cases)
- Missing nodal stage, N=1915
- Nodal stage N3 or tumour stage T3, N=3891
- No surgery date, N=5
- Surgery date prior to 2008, N=3

**INCLUSION CRITERIA:**
**Women with first recorded breast cancer diagnosed 2008-2017, T1-2, N0-2, with known surgery date and type of locoregional treatment**
**N=51423**

- 1 recorded death prior to diagnosis date
- 46 reused personal identification numbers (no register linkage possible)
- 2390 women treated with breast-conserving surgery without adjuvant RT

**Included in analysis**
**N=48986**

© 2021 de Boniface J et al. *JAMA Surgery.*

**eFigure 2. Survival proportions for breast cancer-specific survival by locoregional treatment group and age.**

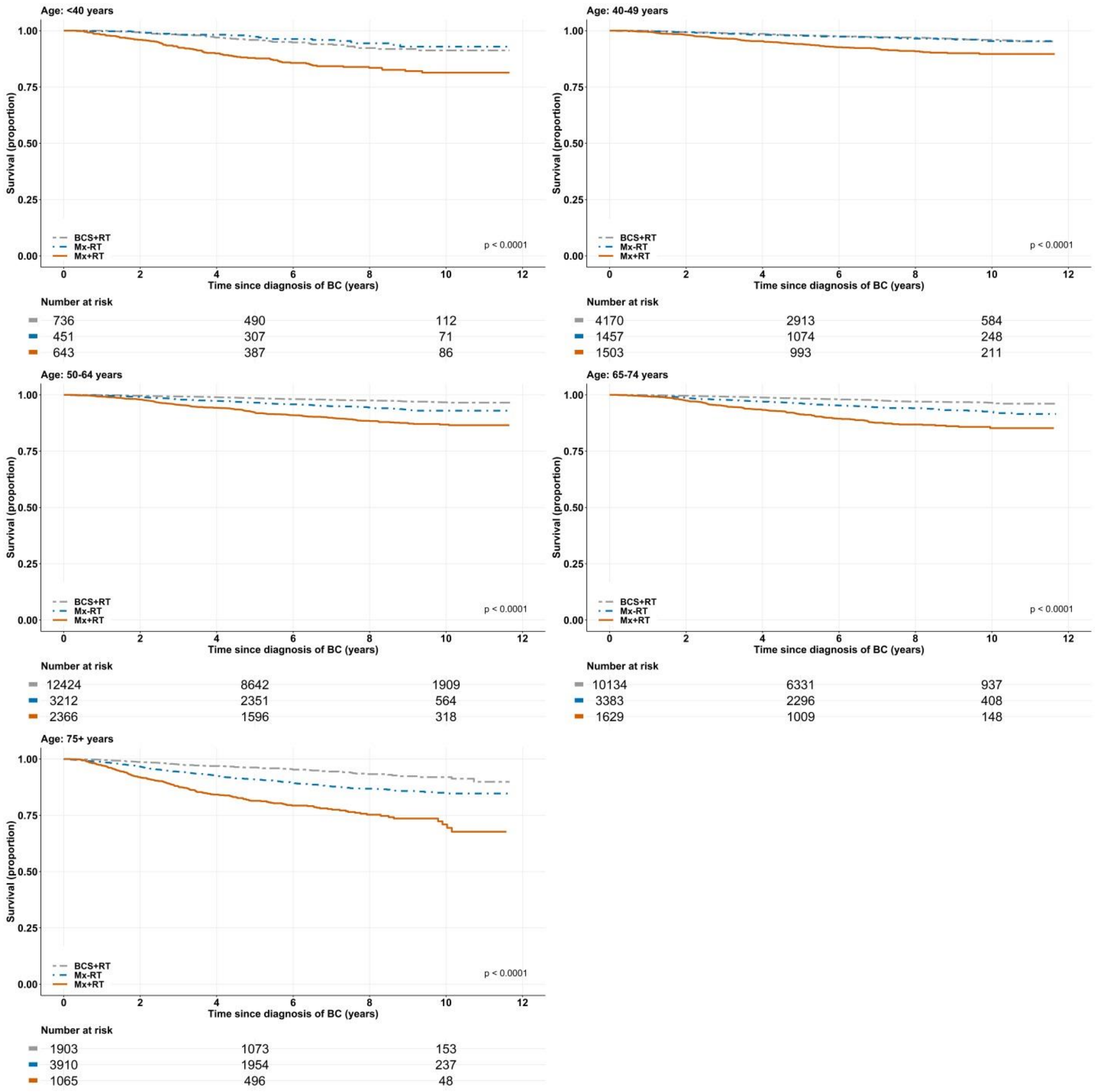


© 2021 de Boniface J et al. *JAMA Surgery.*

**eFigure 3. Survival proportions for overall survival by locoregional treatment group and prognostic group.**

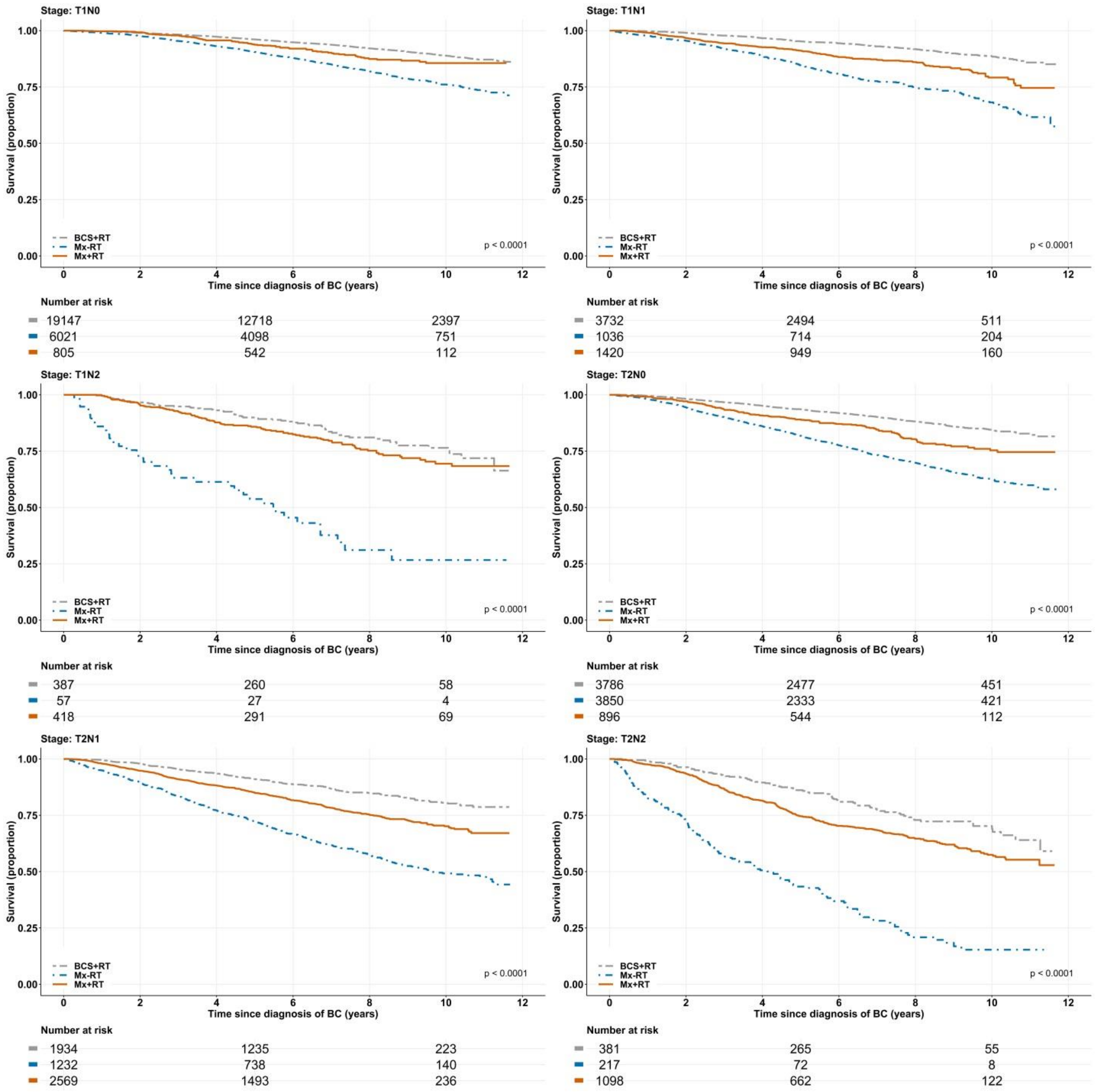


© 2021 de Boniface J et al. *JAMA Surgery.*

**eFigure 4. Survival proportions for overall survival by locoregional treatment group and age.**

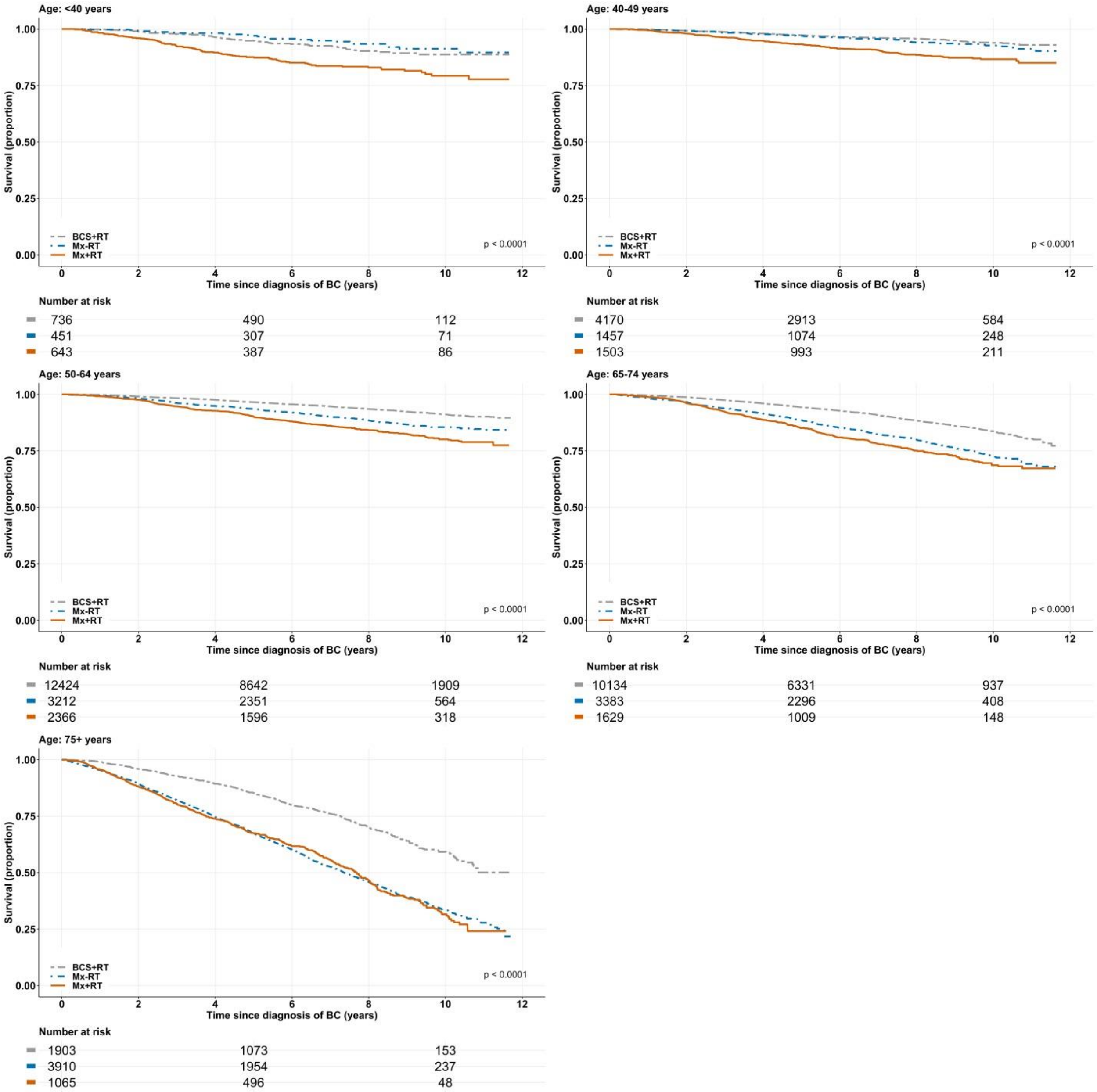


© 2021 de Boniface J et al. *JAMA Surgery.*
